# Impact of prognostic scores on acute kidney injury assessment in the postoperative period of myocardial revascularization

**DOI:** 10.1590/1980-220X-REEUSP-2024-0410en

**Published:** 2025-04-14

**Authors:** Abraão Alves dos Reis, Tayse Tâmara da Paixão Duarte, Michelle Zampieri Ipolito, Kamilla Grasielle Nunes da Silva, Paulo Percio Mota Magro, Marcia Cristina da Silva Magro

**Affiliations:** 1 Universidade de Brasília, Faculdade de Ciências e Tecnologias em Saúde, Brasília, DF, Brazil.; 2 Hospital Universitário de Brasília, Brasília, DF, Brazil.; 3 Instituto Federal de Brasília, Brasília, DF, Brazil.

**Keywords:** Critical Care, Simplified Acute Physiology Score, Acute Kidney Injury, Myocardial Revascularization

## Abstract

**Objective:**

To determine the impact of the Simplified Acute Physiology Score 3 and the Sequential Organ Failure Assessment in assessing the severity of acute kidney injury in patients after cardiopulmonary bypass.

**Method:**

A retrospective cohort study with a non-probabilistic sample. Inferential analysis was performed using Pearson’s chi-square, Fisher’s exact and Mann-Whitney tests, with a significance level of 5%.

**Results:**

The prevalence of acute kidney injury was 31.4%. The Simplified Acute Physiology Score 3 and the Sequential Organ Failure Assessment showed higher scores in patients with kidney injury (58 (48–64) versus 48 (37–57), p = 0.02; 7 (6–9) versus 6 (5–7), p = 0.003), in addition to a longer stay in intensive care, 8 (6–16) versus 6 (5–8) (p = 0.02) days, respectively.

**Conclusion:**

Patients with acute kidney injury remained in intensive care longer, and the Simplified Acute Physiology Score 3 and the Sequential Organ Failure Assessment showed good performance, evidencing greater severity among patients with acute kidney injury in the postoperative period of coronary artery bypass grafting.

## INTRODUCTION

Acute kidney injury (AKI) is a complication after cardiac surgery with incidences between 5% and 42%, depending on the severity, the diagnostic criteria used and the characteristics of patients, which affects short and long-term results^(1)^. The severity of AKI is the main determinant of short- and long-term mortality, with rates of 36.67% and 64.66%^(2)^. Moreover, AKI is associated with increased length of hospital stay and total health-related costs^(3)^.

The pathophysiology of AKI associated with cardiac surgery is not yet fully understood. Generally, multifactorial causes predispose its occurrence during the preoperative, intraoperative and postoperative periods, as it involves complex interactions between patient risk factors, surgical characteristics and subsequent clinical events^(4,5)^. These factors act synergistically and contribute to the development of different mechanisms of kidney injury. Thus, precision of care is crucial, based on the prediction and personalization of patient-centered strategies, implemented precisely at the right time, during the course of clinical management, to achieve better clinical outcomes^(3)^. This includes early identification of individual risk factors, such as pre-existing comorbidities and altered laboratory parameters as well as the adoption of targeted interventions^(4)^.

Coronary artery bypass grafting (CABG) is the predominant cardiac surgery procedure involving cardiopulmonary bypass (CPB). It presents a high risk for the development of AKI, although different interventional approaches have an impact on the onset of AKI^(4)^. This risk occurs due to the complex interaction between the invasive nature of the procedure, the physiological factors involved in CPB, and the clinical characteristics of patients^(6)^.

To adequately manage AKI associated with cardiac surgery, prediction models or scoring systems have been used which, by proving promising in the early identification of patients at risk, facilitate and optimize treatment strategies in the postoperative period and, therefore, can contribute to improving prognosis^(4)^. The prognosis of patients with AKI depends not only on the etiology of the disease that caused it, but also on the diagnostic methods and management used. The use of prognostic scores, such as the Simplified Acute Physiology Score 3 (SAPS 3)^(7)^ and the Sequential Organ Failure Assessment (SOFA)^(8)^, used in the Intensive Care Unit (ICU) to predict the risk of mortality, allows a reasonable estimate of the risk of death for patient-centered clinical decision-making, reducing the possibility of a complicated evolution and worse outcomes.

The efficacy of these prognostic scores appears to be well accepted in intensive care patients; however, there is still a gap when it comes to studies that demonstrate the relationship between SAPS 3 and SOFA in the postoperative period of CABG. The comparative performance between SAPS 3 and SOFA scores in identifying patients at higher risk of unfavorable outcomes remains little explored. In this perspective, this study aimed to determine the impact of using SAPS 3 and SOFA prognostic scores in assessing the severity of patients with AKI in the postoperative period of myocardial revascularization with CPB.

## METHOD

### Study Design, Period and Place

This is a quantitative, retrospective cohort study, conducted based on STrengthening the Reporting of OBservational studies in Epidemiology (STROBE) guidelines. It was conducted in an adult ICU of a public teaching hospital in midwestern Brazil. Data collection was carried out between July 2023 and June 2024.

#### Sample Definition and Selection Criteria

The sample was a non-probabilistic convenience sample, with patients who underwent myocardial revascularization with CPB. Patients over 18 years of age and postoperative of CPB were included. Patients who underwent combined surgeries, palliative care, reoperations, with insufficient data in patients’ medical records, estimated glomerular filtration rate (GFR) below 30 mL/min/1.73 m^2^, according to the Chronic Kidney Disease Epidemiology Collaboration (CKD-EPI) formula^(9)^, or were undergoing treatment for chronic kidney disease, such as dialysis in the pre-hospitalization period or kidney transplantation, were excluded. Of the 87 patients, ten were excluded due to a history of chronic kidney disease, and three due to insufficient information for analysis, thus constituting a sample of 74 patients.

### Study Protocol

Data were collected from patients who underwent on-pump CABG between January 2021 and December 2023. Patients were followed up for 15 days from ICU admission, from the immediate postoperative period and every ten days thereafter until hospital outcome. A questionnaire with structured questions containing clinical and surgical characterization items, prognostic scores (SAPS 3 and SOFA), comorbidities, laboratory, hemodynamic and surgical parameters (CPB time, surgery time, transfusion) was used for data collection. Data extraction was performed from physical and electronic medical records. The SAPS 3 score was recorded upon ICU admission, and the daily SOFA score was recorded for prognostic assessment and identification of the outcome.

Patients included were classified as having or not having AKI in the postoperative period. Baseline creatinine was defined as the lowest creatinine recorded in medical records from seven to 365 days before hospital admission. When absent, the lowest creatinine of the first seven days of hospital admission was recorded^(10)^.

AKI was defined according to the Kidney Disease: Improving Global Outcomes (KDIGO) classification, classified as: KDIGO 1 (risk for renal injury), in the presence of an increase in serum creatinine (SCr) of 0.3 mg/dL in 48 hours or an increase of 1.5 times or 1.9 times the baseline value in seven days; KDIGO 2 (renal injury), when the increase in SCr was 2.0 to 2.9 times the baseline value; and KDIGO 3 (renal failure), when the increase in SCr was 3.0 times the baseline value or when there was a need for renal replacement therapy (RRT). Only patients classified as stage 3 are indicated to start renal replacement therapy. They were classified as having no renal impairment if SCr and previous GFR were greater than or equal to 60 mL/min/1.73 m^2(11)^.

The SAPS 3 and SOFA prognostic prediction systems have proven their use in the clinical setting of critically ill patients admitted to the ICU. SAPS 3 was used because it is a prognostic score for disease severity, with the purpose of predicting mortality based on data obtained at admission. SOFA was used to identify organ dysfunction, describing physiological changes by organ system^(7,8)^. AKI was considered early when patients developed AKI within 48 hours after admission to the ICU, and late AKI occurred when AKI affected patients for periods longer than 48 hours^(12)^.

#### Data Analysis and Treatment

The data were organized in Microsoft Excel 2021^®^ spreadsheets with double checking. Statistical analysis was performed using the Statistical Package for the Social Sciences (IBM^®^SPSS^®^) version 23. There was descriptive analysis with calculation of absolute and relative frequencies, mean, median, standard deviation, and interquartile range to describe the characteristics of variables and provide summary information about the data collected.

For inferential analysis, the Kolmogorov-Smirnov normality test was applied, which did not show normal distribution. Nonparametric tests, such as Pearson’s chi-square test and Fisher’s exact test, were then performed to investigate the association between different categorical variables. The Mann-Whitney test was used to compare the medians of two independent samples in situations where the data did not meet the assumptions of normal distribution and homogeneity of variances. The significance level adopted was 5%.

#### Ethical Aspects

The study was conducted in accordance with national and international ethics guidelines, and was approved by the Research Ethics Committee of the *Universidade de Brasília* School of Health Sciences and Technologies, under Certificate of Presentation for Ethical Consideration 61486022.0.0000.8093 and Opinion 5.740.310. All patients included signed the Informed Consent Form.

## RESULTS

Of the 74 patients with and without AKI, males (77.0%) and brown ethnicity (67.6%) predominated. Age over 60 years was similar between both groups, as was the Body Mass Index greater than 26 kg/m^2^. The majority (74.3%) reported hypertension and diabetes mellitus (43.2%) as comorbidities. All required antibiotics and vasoactive drugs, with norepinephrine being the most frequent (81.1%). In addition, 44.6% used diuretics and 4.1% received RRT.

Almost all (94.6%) were discharged from the ICU. The median length of ICU stay in the group with AKI was slightly longer when compared to the group without AKI (8 (6–16) versus 6 (5–8) days), as was length of hospital stay (22 (16–35) versus 16 (12–32) days). The median length of surgery was over 300 minutes, and the median length of CPB was over 100 minutes in both groups. The severity of AKI patients represented by SAPS 3 was 58 (48–64), and by SOFA, it was 7 (6–9), higher than the group without AKI (Table 1).

**Table 1:** Demographic and clinical characterization of patients in the postoperative period of myocardial revascularization with cardiopulmonary bypass – Brasília, FD, Brazil, 2021–2023.

Characteristics	Without AKI (n = 51)	With AKI (n = 23)	Total (n = 74)
Male sex	41 (80.4)	16 (69.6)	57 (77.0)
Age (years) mean ± SD; median (IQR)	60 ± 8	64 ± 7	62 ± 8
	63 (55–65)	64 (61–70)	63 (57–67)
Ethnicity			
White n (%)	4 (7.8)	5 (21.7)	9 (12.2)
Black n (%)	3 (5.9)	1 (4.3)	4 (5.4)
Yellow n (%)	1 (2.0)	0 (0.0)	1 (1.4)
Brown n (%)	37 (72.5)	13 (56.5)	50 (67.6)
Not declared n (%)	7 (13.7)	3 (13.0)	10 (13.5)
Comorbidities			
Diabetes n (%)	19 (37.3)	13 (56.5)	32 (43.2)
Hypertension n (%)	36 (70.6)	19 (82.6)	55 (74.3)
Respiratory disease in (%)	3 (5.9)	5 (21.7)	8 (10.8)
Peripheral vascular disease n (%)	2 (4.0)	2 (8.7)	4 (5.4)
Cerebrovascular disease n (%)	1 (2.0)	1 (4.3)	2 (2.7)
Medications n (%)			
Used vasoactive drugs	51 (100.0)	23 (100.0)	74 (100.0)
Adrenaline	4 (7.8)	5 (21.7)	9 (12.2)
Dobutamine	49 (96.1)	23 (100.0)	72 (97.3)
Noradrenaline	40 (78.4)	20 (87.0)	60 (81.1)
Vasopressin	11 (21.6)	4 (17.4)	15 (20.3)
Vasodilators	35 (68.6)	13 (56.5)	48 (64.9)
Used ATB	51 (100.0)	23 (100.0)	74 (100.0)
Polymyxin	0 (0.0)	1 (4.3)	1 (1.4)
Glycopeptide ATB	2 (3.9)	7 (30.4)	9 (12.2)
Aminoglycoside ATB	1 (2.0)	3 (13.0)	4 (5.4)
Antifungal ATB	0 (0.0)	1 (4.3)	1 (1.4)
Used diuretic	20 (39.2)	13 (56.5)	33 (44.6)
Loop diuretic	18 (35.3)	13 (56.5)	31 (41.9)
Potassium-sparing diuretic	4 (7.8)	2 (8.7)	6 (8.1)
RRT n (%)	0 (0.0)	3 (13.0)	3 (4.1)
ICU outcome n (%)			
Discharge for outpatient care	48 (94.1)	22 (95.7)	70 (94.6)
Hospital discharge	2 (4.0)	2 (4.0)	4 (5.4%)
Hospital outcome n (%)			
Death	1 (2.0)	2 (8.7)	3 (4.1)
Discharged home	34 (66.7)	11 (47.8)	45 (60.8)
Referred to another department	16 (31.4)	8 (34.8)	24 (32.4)
Referred to another hospital	0 (0.0)	1 (4.3)	1 (1.4)
Length of ICU stay (days) median (IQR)	6 (5–8)	8 (6-16)	7 (5-9)
Length of hospital stay (days) median (IQR)	16 (12-32)	22 (16–35)	20 (13–32)
Length of surgery (minutes) median (IQR)	330 (300–360)	360 (315–405)	330 (300–360)
Length of CPB (minutes) median (IQR)	101 (80–120)	112 (101–142)	105 (90–120)
SAPS 3 (admission) median (IQR)	48 (37–57)	58 (48–64)	53 (41–60)
Maximum SOFA median (IQR)	6 (5–7)	7 (6–9)	6 (5–7)

Legend: ATB – antibiotic; CPB – cardiopulmonary bypass; BMI – Body Mass Index; AKI – acute kidney injury; SAPS 3 – Simplified Acute Physiology Score 3; SOFA – Sequential Organ Failure Assessment; ICU – Intensive Care Unit; IQR – interquartile range; RRT – renal replacement therapy; SD – standard deviation.

In the group of patients with AKI, KDIGO 1 (12.2%) and 2 (12.2%) predominated, i.e., with risk and renal injury. However, more than half presented preserved renal function (68.9%). Of the total of 23 patients with AKI, 20 (87.0%) were diagnosed early, and three (13.0%) were diagnosed late.

Urea was higher in the group of patients with AKI (43 (37–66) mg/dL), as was creatinine (1.00 (0.85–1.35)). Both were significantly different between the groups, as was pH (p-value = 0.001 versus 0.03). Hematocrit and hemoglobin were significantly reduced in the group with AKI (p-value < 0.001 versus 0.001). The other biochemical and hemodynamic parameters were similar and within the tolerable range of normality during patients’ evolution period (Table 2).

**Table 2:** Hemodynamic and biochemical parameters of patients with and without acute kidney injury – Brasília, FD, Brazil, 2021–2023.

Characteristics	Without AKI (n = 51)	With AKI (n = 23)	Total (n = 74)	p-value
Mean urea (mg/dL) median (IQR)	32 (30–36)	43 (37–66)	35 (30–44)	0.001
Mean pH median (IQR)	7.37 (7.33–7.40)	7.39 (7.36–7.42)	7.37 (7.34–7.40)	0.03
Mean lactate (mg/dL) median (IQR)	26.8 (21.5–39.0)	22.1 (14.8–31.5)	25.8 (18.1–35.0)	0.1
Mean potassium (mEq/L) median (IQR)	4.3 (4.1–4.5)	4.1 (4.0–4.4)	4.2 (4.0–4.4)	0.1
Mean sodium (mEq/L) median (IQR)	139 (138–140)	139 (138–143)	139 (138–141)	0.09
MAP (mmHg) median (IQR)	95 (90–98)	98 (92–103)	96 (90–101)	0.07
Mean heart rate (bpm) median (IQR)	98 (93–104)	103 (95–111)	99 (93–106)	0.1
Respiratory rate (bpm) median (IQR)	20.3 (18.9–21.8)	20.7 (19.9–22.4)	20.5 (19.0–22.0)	0.3
Mean SpO2 (%) median (IQR)	96.6 (95.6–96.9)	96.8 (95.6–97.3)	96.6 (95.6–97.0)	0.4
Serum creatinine (IQR)	0.87 (0.76–1.01)	1.00 (0.85–1.35)	0.90 (0.78–1.05)	0.046
Mean Hb (g/dL) median (IQR)	10.4 (9.7–11.0)	9.4 (9.0–10.1)	10.1 (9.2–10.7)	<0.001
Mean Ht (%) median (IQR)	30.5 (28.4–32.5)	27.7 (26.3–29.9)	29.8 (27.5–31.8)	0.001
Glucose (mg/dL) median (IQR)	187 (156–224)	192 (165–230)	188 (160–225)	0.4
Mean platelet (μL) median (IQR)	199000 (174000–245000)	217000 (169000–263000)	205000 (170000–255000)	0.7

Legend: Mann-Whitney U test; Hb – hemoglobin; Ht – hematocrit; MAP – mean blood pressure; AKI – acute kidney injury; IQR – interquartile range; SpO2 – oxygen saturation.

As shown in Table 3, regardless of the group, males were predominant. The median age of over 60 years characterized both groups, as well as overweight and black or brown, although without statistical difference. In more than half of both groups, the reason for hospitalization was surgery. Beta-lactam antibiotics, such as cefuroxime, cefazolin and piperacillin, were used significantly. The length of stay in the ICU was significantly higher in the group with AKI (p-value = 0.02), as was the length of CPB (p-value = 0.04). SAPS 3 and SOFA were significantly altered in the group with AKI (p-value 0.02 versus 0.003).

**Table 3:** Association of clinical variables with the presence or absence of acute kidney injury – Brasília, FD, Brazil, 2021–2023.

Characteristics	Without AKI (n = 51)	With AKI (n = 23)	p-value
Male sex	41 (80.4)	16 (69.6)	0.3
Age (years) median (IQR)	63 (55–65)	64 (61–70)	0.1
BMI (kg/m^2^) median (IQR)	26.5 (24.7–28.3)	26.9 (23.6–29.6)	0.9
Ethnicity			
Black or brown n (%)	37 (72.5)	14 (60.9)	0.1
Comorbidities n (%)			
Diabetes mellitus	19 (37.3)	13 (56.5)	0.2
Hypertension	36 (70.6)	19 (82.6)	0.3
Respiratory disease	3 (5.9)	5 (21.7)	0.06
Peripheral vascular disease	2 (4.0)	2 (8.7)	0.6
Cerebrovascular disease	1 (2.0)	1 (4.3)	0.5
Medications n (%)			
Catecholamine	50 (98.0)	23 (100.0)	0.9
Adrenaline	4 (7.8)	5 (21.7)	0.1
Dobutamine	49 (96.1)	23 (100.0)	0.9
Noradrenaline	40 (78.4)	20 (87.0)	0.5
Vasopressin	11 (21.6)	4 (17.4)	0.8
Vasodilators	35 (68.6)	13 (56.5)	0.3
Cefazolin	32 (66.7)	19 (90.5)	0.03
Cefuroxime	16 (31.4)	2 (8.7)	0.03
Meropenem	2 (3.9)	4 (17.4)	0.07
Piperacillin	4 (7.8)	7 (30.4)	0.01
Polymyxin	0 (0.0)	1 (4.3)	0.3
Glycopeptide ATB	2 (3.9)	7 (30.4)	0.003
Aminoglycoside ATB	1 (2.0)	3 (13.0)	0.09
Antifungal ATB	0 (0.0)	1 (4.3)	0.3
Used diuretic	20 (39.2)	13 (56.5)	0.2
RRT n (%)	0 (0.0)	3 (13.0)	0.07
ICU outcome n (%)
Discharge for outpatient care	48 (94.1)	22 (95.7)	0.9
Final outcome n (%)
Discharged home	34 (66.7)	11 (47.8)	0.1
Length of ICU stay (days) median (IQR)	6 (5–8)	8 (6–16)	0.02
Length of hospital stay (days) median (IQR)	16 (12–32)	22 (16–35)	0.2
Length of surgery (minutes) median (IQR)	330 (300–360)	360 (315–405)	0.2
Length of CPB (minutes) median (IQR)	101 (80–120)	112 (101–142)	0.04
SAPS3 (admission) median (IQR)	48 (37–57)	58 (48–64)	0.02
Maximum SOFA median (IQR)	6 (5–7)	7 (6–9)	0.003

Legend: Fisher’s exact test; chi-square test; Mann-Whitney U test; ATB – antibiotic; CPB – cardiopulmonary bypass; BMI – Body Mass Index; AKI – acute kidney injury; ICU – Intensive Care Unit; IQR – interquartile range; RRT – renal replacement therapy.

**Figure 1:**
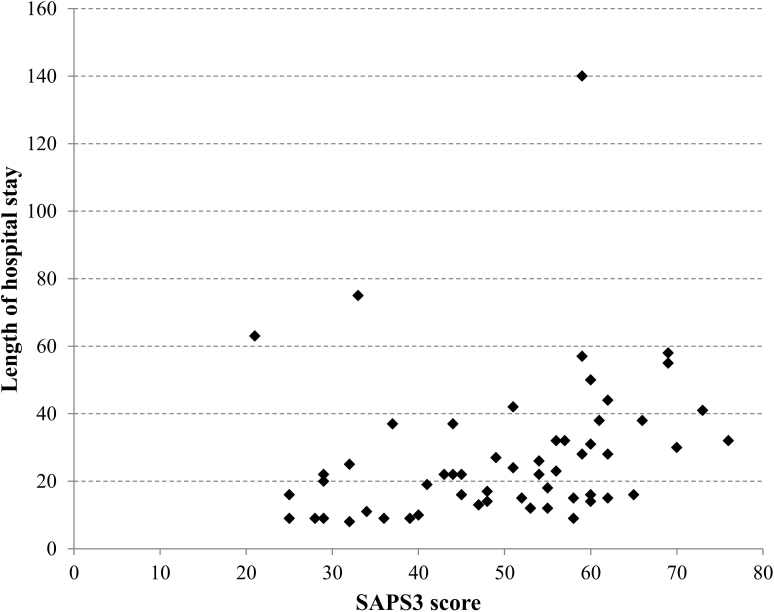
Correlation between SAPS 3 score and length of hospital stay (Rho = 0.402; p-value = 0.002). Brasília, FD, Brazil, 2021–2023.

Figure 1 shows that increasing the length of hospital stay also increases the severity of patients’ condition, according to the SAPS 3 score.

**Figure 2:**
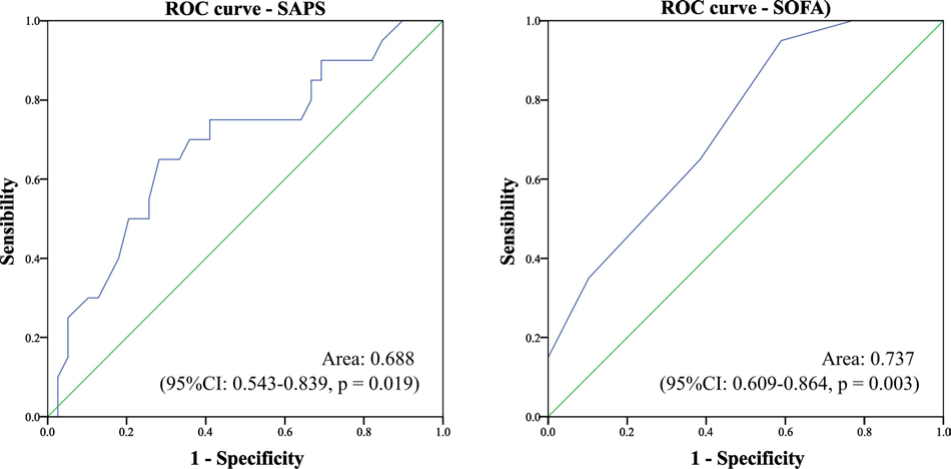
Correlation between SAPS 3 and SOFA scores and acute kidney injury. Brasília, FD, Brazil, 2021–2023.

Figure 2 shows that both SAPS 3 and SOFA stood out for their significant association with AKI, with a good area under the curve of 0.688 versus 0.737.

## DISCUSSION

The results of this study showed that, in the postoperative period of myocardial revascularization with CPB, approximately one in three patients developed mild and moderate AKI (KDIGO 1 and 2). The impact of SAPS 3 and SOFA scores on the clinical assessment of the group with AKI showed high severity, when compared to the group without AKI, although in-hospital mortality affected approximately one in 12 patients and the predominant ICU and hospital outcomes were discharge, supported by the low need for RRT and the permanence of several biochemical and hemodynamic indicators, in general, within normal limits in the population studied, despite the length of hospital stay being prolonged and associated with greater clinical severity and consequent risk of death in the long term.

Results of scientific evidence have shown that, even in mild and reversible AKI, as evidenced in the present investigation, there is a continuous risk of tissue and organic damage and that, *a priori*, severe AKI may represent an irreversible decrease in renal function and even end-stage renal failure^(13,14)^. Studies show a higher probability of AKI in older adults with heart failure and a history of hypertension, diabetes and other diseases, in line with the findings of this study. High expressions of blood urea nitrogen and SCr, in addition to low expression of estimated GFR, indicate impaired renal function after surgery and, therefore, a higher risk of AKI, even if of mild and moderate severity(14).

Although the mean age of patients with AKI globally is 60 years, with decreasing socioeconomic status, this age can be reduced to around 50 years in low- and middle-income countries. In this study, the mean age of patients with AKI was higher than that of the group without AKI, suggesting that advanced age increases the risk of AKI after CABG^(15,16)^, supporting the fact that the majority were male, given that scientific evidence highlights that 60% of patients with AKI are men^(16,17)^.

On-pump CABG has been associated with significant increases in total costs, postoperative costs, length of ICU stay, and postoperative hospital stay. Therefore, predicting these patients’ future health status is crucial. Identifying changes in their clinical situation as early as possible can contribute to treatment adjustment and, therefore, prevention of organ failure with unfavorable outcomes, such as death^(18)^. In this study, the longer the hospital stay, the greater the severity of patients’ condition according to the SAPS 3 and SOFA prognostic scores.

Despite scientific advances, cardiovascular surgery still involves a risk of serious complications in the critical care setting. AKI represents a notable complication, with a significant impact on patients’ clinical evolution, determining a high incidence of morbidity and mortality^(19)^. Furthermore, AKI requires RRT in 2% to 5% of affected patients, a condition that can further worsen mortality^(19)^. In this study, approximately one in eight patients required RRT, although the majority were discharged home.

Due to the morbidity and mortality associated with worsening renal function, early diagnosis is important and should be sought in at-risk populations^(19)^, such as older adults, also present in most of this study. In a retrospective cohort study conducted in a Chinese population, the in-hospital mortality rates for patients without AKI and with AKI stages 1, 2, and 3 were 0%, 2.4%, 10.0%, and 22.2%^(15)^, respectively, similar to the findings of this investigation.

After surgery, AKI may also affect the prognosis and reduce the long-term survival rate of patients^(15,16)^. Among the findings of this study, most patients with AKI in the postoperative period of revascularization with CPB were identified early, i.e., in the first 48 hours after surgery, which highlights the importance of electronic alerts in laboratory programs as a trigger mechanism for early detection of AKI. In clinical practice, by recognizing changes in SCr, these alerts increase notification and provide earlier initiatives. With this, it is possible to obtain better results for patients, as well as potential benefits during hospitalization and in the long term^(15,20)^.

A retrospective study conducted with data from patients undergoing open-heart surgery in Saudi Arabia found that patients with CPB times longer than 90 minutes had more significant kidney damage^(21)^. The results of this study showed that CPB time longer than 100 minutes significantly impacted renal impairment. CPB can produce a series of inflammatory factors and cause tissue damage. Prolonged CPB support can increase hemolysis, release of free hemoglobin, and impair renal function by releasing iron as an endogenous toxin^(15)^.

CPB alone or associated with aging may predispose to reduced renal mass, tubular and sclerotic alteration of the kidney. This process causes reduced renal blood flow and a decline in GFR at a rate of 6.3 mL/min/1.73 m^2^ per decade. Therefore, decreased physiological capacity of the kidney increases the risk of AKI^(19)^. In this study, the sample was characterized mainly by older adult patients, although age alone was not a significant factor for the occurrence of AKI.

A systematic review of 13 studies showed that the median length of stay in the ICU for patients with AKI according to the KDIGO creatinine criterion was 0.75 to 10 days^(22)^, similar to the findings of this study, in which the median length of hospital stay in this group of patients was eight days.

CABG imposes the need for intraoperative red blood cell transfusion, as highlighted in a retrospective cohort study^(15)^. In the present study, it was possible to identify a significant difference in hemoglobin levels between patients with and without AKI. Decreased hemoglobin levels are determining factors for transfusion^(23)^. Both anemia and red blood transfusion may increase the risk of perioperative AKI. Anemia causes increased renal hypoxic stress and the need for red blood transfusion by increasing the inflammatory response and free radical-related injury^(23)^.

Meta-analysis of clinical trials found that patients with AKI are characterized by rapid loss of renal function, which can trigger electrolyte disturbances, metabolic acidosis, fluid overload, and increased serum uremic toxins^(24)^. Uremia is a pathological process that accompanies AKI^(24)^, as identified among AKI patients in this study. Renal and cardiac diseases are interrelated, and in both disorders, several common risk factors are observed, such as arterial hypertension and diabetes mellitus, also recognized among the findings of this study as the most frequent comorbidities.

The establishment of prognostic scores, such as SOFA and SAPS 3, based on systemic functions of multiple organs, is a strategy for analyzing the prognostic severity of critically ill patients and one of the best ways to reduce risks, supporting patient-centered care^(25)^. It is widely used in intensive care to guide clinical decisions and care^(26)^. As evidenced in these findings, with a good area under the curve, prognostic scores proved to be reliable predictors of AKI in the postoperative period of myocardial revascularization with CPB.

A systematic review of 15 studies concluded that renal toxicity was greater in patients receiving vancomycin combined with piperacillin-tazobactam than with any other agent alone or vancomycin combined with meropenem or cefepime. The present findings showed that beta-lactam antibiotics, such as those described, were significantly used in patients who developed AKI in the postoperative period of CABG. The use of nephrotoxic drugs is a modifiable risk factor for AKI and contributes to approximately 18.2% to 48.7% of AKI cases in critically ill patients, in whom antibiotics act as essential triggers for AKI^(27,28)^.

Treatment options for AKI are very limited. Clinical diagnosis and recognition of AKI signs by professionals such as nurses can contribute to the implementation of appropriate preventive measures, based on scoring systems/prognostic scores, which can effectively favor early recovery from AKI, even if the diagnosis of this syndrome is clinically late and guided by SCr.

The limitations of this study result from the single-center approach, with a risk of measurement bias related to data recording in medical records, limited sample size and use only of the creatinine criterion of the KDIGO classification, to assess renal function, due to the inaccuracy of the urinary volume recording.

## CONCLUSION

Prognostic scores such as SAPS 3 and SOFA showed good performance and showed that the group with AKI was clinically more severe. Among patients in the postoperative period of CABG, approximately one in three developed AKI of mild and moderate severity (KDIGO 1 and 2) and a longer stay in both the ICU and the hospital unit.
