# Supplementary material for: Impact of prognostic scores on acute kidney injury assessment in the postoperative period of myocardial revascularization
Source: Rev Esc Enferm USP. 2025 Apr 14;59:e20240410. doi: 10.1590/1980-220X-REEUSP-2024-0410en (PMC11999537; doi:10.1590/1980-220X-REEUSP-2024-0410en)
Supplement: Translation

# Translation: Impacto de escores prognósticos na avaliação da injúria renal aguda em pós-operatório de revascularização do miocárdio

**DOI:** 10.1590/1980-220X-REEUSP-2024-0410pt

**Authors:** Abraão Alves dos Reis, Tayse Tâmara da Paixão Duarte, Michelle Zampieri Ipolito, Kamilla Grasielle Nunes da Silva, Paulo Percio Mota Magro, Marcia Cristina da Silva Magro

**Affiliations:** 1 Universidade de Brasília, Faculdade de Ciências e Tecnologias em Saúde, Brasília, DF, Brasil.; 2 Hospital Universitário de Brasília, Brasília, DF, Brasil.; 3 Instituto Federal de Brasília, Brasília, DF, Brasil.

**Keywords:** Cuidados Críticos, Escore Fisiológico Agudo Simplificado, Injúria Renal Aguda, Revascularização Miocárdica

## Abstract

**Objetivo:**

Determinar o impacto do *Simplified Acute Physiology Score 3* e do *Sequential Organ Failure Assessment* na avaliação da gravidade de pacientes com injúria renal aguda no pós-operatório de revascularização do miocárdio com circulação extracorpórea.

**Método:**

Estudo de coorte retrospectiva, com amostra não probabilística. Realizou-se análise inferencial com testes qui-quadrado de Pearson, exato de Fisher e Mann-Whitney, com nível de significância de 5%.

**Resultados:**

A prevalência de injúria renal aguda foi de 31,4%. O *Simplified Acute Physiology Score 3* e o *Sequential Organ Failure Assessment* apresentaram pontuações maiores nos pacientes com injúria renal (58 (48–64) *versus* 48 (37–57), p = 0,02; 7 (6–9) *versus* 6 (5–7), p = 0,003), além de maior tempo de permanência na terapia intensiva, de 8 (6–16) *versus* 6 (5–8) (p = 0,02) dias, respectivamente.

**Conclusão:**

Pacientes com injúria renal aguda permaneceram mais tempo na terapia intensiva, e o *Simplified Acute Physiology Score 3* e o *Sequential Organ Failure Assessment* mostraram bom desempenho, evidenciando maior gravidade entre os pacientes com injúria renal aguda no pós-operatório de cirurgia de revascularização do miocárdio.

## INTRODUÇÃO

A injúria renal aguda (IRA) é uma complicação após cirurgia cardíaca com incidências entre 5% e 42%, dependendo da gravidade, dos critérios diagnósticos utilizados e das características dos pacientes, o que afeta os resultados de curto e longo prazo^(1)^. A gravidade da IRA é o principal determinante da mortalidade a curto e longo prazo, com taxas de 36,67% e 64,66%^(2)^. Além disso, a IRA está associada ao aumento do tempo de internação hospitalar e dos custos totais relacionados à saúde^(3)^.

A fisiopatologia da IRA associada à cirurgia cardíaca ainda não é totalmente compreendida. Geralmente, causas multifatoriais predispõem sua ocorrência durante os períodos pré-operatório, intraoperatório e pós-operatório, por envolver interações complexas entre fatores de risco do paciente, características da cirurgia e eventos clínicos subsequentes^(4,5)^. Esses fatores atuam de maneira sinérgica e contribuem para o desenvolvimento de diferentes mecanismos da injúria renal. Assim, a precisão do cuidado é crucial, fundamentada na predição e personalização de estratégias centradas no paciente, implementadas precisamente no momento certo, durante o curso do gerenciamento clínico, para o alcance de melhores resultados clínicos^(3)^. Isso inclui a identificação precoce de fatores de risco individuais, como comorbidades pré-existentes e parâmetros laboratoriais alterados, bem como a adoção de intervenções direcionadas^(4)^.

A cirurgia de revascularização do miocárdio (CRM) predomina entre os procedimentos de cirurgia cardíaca com circulação extracorpórea (CEC). Apresenta grande risco para o desenvolvimento de IRA, embora diferentes abordagens intervencionistas tenham impacto no início da IRA^(4)^. Esse risco ocorre a partir da complexa interação entre a natureza invasiva do procedimento, os fatores fisiológicos envolvidos na CEC e as características clínicas dos pacientes^(6)^.

Para gerenciar adequadamente a IRA associada à cirurgia cardíaca, tem-se utilizado modelos de predição ou sistemas de pontuação que, ao se revelarem promissores na identificação precoce de pacientes em risco, facilitam e otimizam as estratégias de tratamento no pós-operatório e, portanto, podem contribuir para melhorar o prognóstico^(4)^. O prognóstico dos pacientes com IRA não depende apenas da etiologia da doença que a originou, mas também dos métodos diagnósticos e do manejo utilizados. O emprego de escores prognósticos, como o *Simplified Acute Physiology Score* 3 (SAPS 3)^(7)^ e o *Sequential Organ Failure Assessment* (SOFA)^(8)^, utilizados na Unidade de Terapia Intensiva (UTI) para prever o risco de mortalidade, permite uma estimativa razoável do risco de morte para tomada de decisão clínica centrada no paciente, reduzindo a possibilidade de uma evolução complicada e piores desfechos.

A eficácia desses escores prognósticos parece ser bem aceita em pacientes de terapia intensiva, entretanto ainda há uma lacuna quando se tratam de estudos que evidenciam a relação entre SAPS 3 e SOFA no pós-operatório de CRM. O desempenho comparativo entre os escores SAPS 3 e SOFA na identificação de pacientes com maior risco de desfechos desfavoráveis permanece pouco explorado. Nessa perspectiva, o objetivo deste estudo é determinar o impacto do uso dos escores prognósticos SAPS 3 e SOFA na avaliação da gravidade de pacientes com IRA no pós-operatório de revascularização do miocárdio com CEC.

## MÉTODO

### Desenho, Período e Local do Estudo

Trata-se de estudo quantitativo, de coorte retrospectiva, realizado com base na diretriz *STrengthening the Reporting of OBservational studies in Epidemiology* (STROBE). Foi realizado em uma UTI adulto de um hospital público de ensino da região Centro-Oeste, Brasil. A coleta de dados foi realizada entre julho de 2023 e junho de 2024.

### Definição da Amostra e Critérios de Seleção

A amostra foi do tipo não probabilística de conveniência, com pacientes que realizaram revascularização miocárdica com CEC. Foram incluídos pacientes com idade superior a 18 anos e pós-operatório de cirurgia de revascularização com CEC. Foram excluídos os que realizaram cirurgias combinadas, em cuidados paliativos, reoperações, com dados insuficientes no prontuário do paciente, taxa de filtração glomerular (TFG) estimada inferior a 30 mL/min/1,73 m^2^, conforme fórmula *Chronic Kidney Disease Epidemiology Collaboration* (CKD-EPI)^(9)^, ou em tratamento para doença renal crônica, como diálise no período pré-internação ou transplante renal. De 87 pacientes, dez foram excluídos por histórico de doença renal crônica, e três, por informações insuficientes para análise, constituindo, assim, uma amostra de 74 pacientes.

### Protocolo do Estudo

Foram coletados dados de pacientes que realizaram CRM com CEC entre janeiro de 2021 e dezembro de 2023. O seguimento dos pacientes ocorreu durante 15 dias a partir da internação na UTI, desde o período de pós-operatório imediato e após de dez em dez dias até o desfecho hospitalar. Para coleta de dados, foi utilizado questionário com questões estruturadas contendo itens de caracterização clínica e cirúrgica, escores prognósticos (SAPS 3 e SOFA), comorbidades, parâmetros laboratoriais, hemodinâmicos e cirúrgicos (tempo de CEC, tempo de cirurgia, transfusão). A extração dos dados foi realizada a partir de prontuários físicos e eletrônicos. O escore SAPS 3 foi registrado na admissão na UTI, e o SOFA diário, para avaliação prognóstica e identificação do desfecho.

Os pacientes incluídos foram classificados com IRA e sem IRA no pós-operatório. A creatinina basal foi definida como a menor creatinina registrada no prontuário no período de sete até 365 dias antes da internação hospitalar. Quando ausente, registrou-se a menor creatinina dos primeiros sete dias de internação hospitalar^(10)^.

A IRA foi definida segundo a classificação *Kidney Disease: Improving Global Outcomes* (KDIGO), classificada em: KDIGO 1 (risco para injúria renal), na presença de aumento da creatinina sérica (Crs) em 0,3 mg/dL em 48 horas ou aumento de 1,5 vezes ou 1,9 vezes do valor basal em sete dias; KDIGO 2 (injúria renal), quando o aumento da Crs foi 2,0 a 2,9 vezes o valor basal; e KDIGO 3 (falência renal), quando o aumento da Crs foi de 3,0 vezes o valor basal ou quando houve necessidade de terapia de substituição renal (TSR). Somente pacientes classificados como estágio 3 têm indicação para iniciar tratamento de substituição renal. Foram classificados como ausência de agravo renal se a Crs e a TFG anterior se apresentassem maiores ou iguais a 60 mL/min/1,73 m^2(11)^.

Os sistemas de predição prognóstica SAPS 3 e o SOFA têm seu uso comprovado no cenário clínico de pacientes em estado crítico internados em UTI. O SAPS 3 foi utilizado por ser um escore prognóstico para gravidade da doença, com a finalidade preditiva de mortalidade a partir de dados obtidos na admissão. O SOFA foi utilizado para identificar a disfunção orgânica, descrevendo alterações fisiológicas por sistema de órgãos^(7,8)^. A IRA foi considerada precoce quando o paciente evoluiu com IRA em até 48 horas após internação na UTI, e a IRA tardia ocorreu quando a IRA acometeu os pacientes por períodos superiores a 48 horas^(12)^.

### Análise e Tratamento dos Dados

Os dados foram organizados em planilhas do *Microsoft Excel 2021* ^®^ com dupla conferência. A análise estatística foi realizada utilizando o *software Statistical Package for the Social Sciences* (IBM^®^SPSS^®^), versão 23. Houve análise descritiva com cálculo de frequências absolutas e relativas, média, mediana, desvio padrão, e intervalo interquartílico para descrever as características das variáveis e fornecer informações resumidas sobre os dados coletados.

Para análise inferencial, foi aplicado teste de normalidade de Kolmogorov-Smirnov, o qual não mostrou distribuição normal. Foram, então, realizados testes não paramétricos, como o teste qui-quadrado de Pearson e o teste exato de Fisher, para investigar a associação entre diferentes variáveis categóricas. O teste de Mann-Whitney foi empregado para comparar as medianas de duas amostras independentes em situações em que os dados não atendiam aos pressupostos de distribuição normal e homogeneidade de variâncias. O nível de significância adotado foi de 5%.

### Aspectos Éticos

O estudo foi conduzido de acordo com as diretrizes de ética nacionais e internacionais, aprovado pelo Comitê de Ética em Pesquisa com Seres Humanos da Faculdade de Ciências e Tecnologias em Saúde da Universidade de Brasília, sob Certificado de Apresentação para Apreciação Ética n° 61486022.0.0000.8093 e Parecer n° 5.740.310. Todos os pacientes incluídos assinaram o Termo de Consentimento Livre e Esclarecido.

## RESULTADOS

Do total de 74 pacientes com e sem IRA, predominaram o sexo masculino (77,0%) e a etnia parda (67,6%). A idade acima de 60 anos foi similar entre ambos os grupos, assim como o Índice de Massa Corporal superior a 26 kg/m^2^. A maioria (74,3%) informou como comorbidades a hipertensão arterial e diabetes mellitus (43,2%). Todos necessitaram de antibióticos e drogas vasoativas, sendo a noradrenalina a mais frequente (81,1%). Além disso, 44,6% usaram diuréticos e 4,1% receberam TSR.

Quase todos (94,6%) evoluíram com alta da UTI. O tempo mediano de internação na UTI do grupo com IRA foi um pouco maior quando comparado ao sem IRA (8 (6–16) *versus* 6 (5–8) dias), assim como o tempo de internação hospitalar (22 (16–35) *versus* 16 (12–32) dias). O tempo mediano de cirurgia foi superior a 300 minutos, assim como o de CEC foi superior a 100 minutos em ambos os grupos. A gravidade dos pacientes com IRA representada pelo SAPS 3 foi de 58 (48-64), e pelo SOFA, foi de 7 (6–9), superior ao grupo sem IRA (Tabela 1).

**Tabela 1:** Caracterização demográfica e clínica dos pacientes em pós-operatório de revascularização do miocárdio com circulação extracorpórea – Brasília, DF, Brasil, 2021–2023.

Características	Sem LRA (n = 51)	Com LRA (n = 23)	Total (n = 74)
Sexo masculino	41 (80,4)	16 (69,6)	57 (77,0)
Idade (anos) média±DP; mediana (IIQ)	60 ± 8	64 ± 7	62 ± 8
	63 (55–65)	64 (61–70)	63 (57–67)
Etnia			
Branca n (%)	4 (7,8)	5 (21,7)	9 (12,2)
Negra n (%)	3 (5,9)	1 (4,3)	4 (5,4)
Amarela n (%)	1 (2,0)	0 (0,0)	1 (1,4)
Parda n (%)	37 (72,5)	13 (56,5)	50 (67,6)
Não declarada n (%)	7 (13,7)	3 (13,0)	10 (13,5)
Comorbidades			
Diabetes n (%)	19 (37,3)	13 (56,5)	32 (43,2)
Hipertensão n (%)	36 (70,6)	19 (82,6)	55 (74,3)
Doença respiratória n (%)	3 (5,9)	5 (21,7)	8 (10,8)
Doença vascular periférica n (%)	2 (4,0)	2 (8,7)	4 (5,4)
Doença vascular cerebral n (%)	1 (2,0)	1 (4,3)	2 (2,7)
Medicamentos n (%)			
Usou drogas vasoativas	51 (100,0)	23 (100,0)	74 (100,0)
Adrenalina	4 (7,8)	5 (21,7)	9 (12,2)
Dobutamina	49 (96,1)	23 (100,0)	72 (97,3)
Noradrenalina	40 (78,4)	20 (87,0)	60 (81,1)
Vasopressina	11 (21,6)	4 (17,4)	15 (20,3)
Vasodilatadores	35 (68,6)	13 (56,5)	48 (64,9)
Usou ATB	51 (100,0)	23 (100,0)	74 (100,0)
Polimixina	0 (0,0)	1 (4,3)	1 (1,4)
ATB glicopeptídeo	2 (3,9)	7 (30,4)	9 (12,2)
ATB aminoglicosídeo	1 (2,0)	3 (13,0)	4 (5,4)
ATB antifúngico	0 (0,0)	1 (4,3)	1 (1,4)
Usou diurético	20 (39,2)	13 (56,5)	33 (44,6)
Diurético de alça	18 (35,3)	13 (56,5)	31 (41,9)
Diurético poupador de potássio	4 (7,8)	2 (8,7)	6 (8,1)
TSR n (%)	0 (0,0)	3 (13,0)	3 (4,1)
Desfecho da UTI n (%)			
Alta para enfermaria	48 (94,1)	22 (95,7)	70 (94,6)
Alta hospitalar	2 (4,0)	2 (4,0)	4 (5,4%)
Desfecho hospitalar n (%)			
Óbito	1 (2,0)	2 (8,7)	3 (4,1)
Alta para casa	34 (66,7)	11 (47,8)	45 (60,8)
Encaminhado para outro setor	16 (31,4)	8 (34,8)	24 (32,4)
Encaminhado para outro hospital	0 (0,0)	1 (4,3)	1 (1,4)
Tempo de UTI (dias) mediana (IIQ)	6 (5–8)	8 (6-16)	7 (5-9)
Tempo de hospital (dias) mediana (IIQ)	16 (12-32)	22 (16–35)	20 (13–32)
Tempo de cirurgia (minutos) mediana (IIQ)	330 (300–360)	360 (315–405)	330 (300–360)
Tempo de CEC (minutos) mediana (IIQ)	101 (80–120)	112 (101–142)	105 (90–120)
SAPS 3 (admissão) mediana (IIQ)	48 (37–57)	58 (48–64)	53 (41–60)
SOFA máximo mediana (IIQ)	6 (5–7)	7 (6–9)	6 (5–7)

Legenda: ATB – antibiótico; CEC – circulação extracorpórea; IMC – Índice de Massa Corporal; IRA – injúria renal aguda; SAPS 3 – *Simplified Acute Physiology Score 3*; SOFA – *Sequential Organ Failure Assessment*; UTI – Unidade de Terapia Intensiva; IIQ – intervalo interquartil; TSR – terapia de substituição renal; DP – desvio padrão.

No grupo de pacientes com IRA, predominaram KDIGO 1 (12,2%) e 2 (12,2%), ou seja, com risco e injúria renal. No entanto, mais da metade apresentou função renal preservada (68,9%). Do total de 23 pacientes com IRA, 20 (87,0%) foram diagnosticadas precocemente, e três (13,0%), tardiamente.

A ureia foi superior no grupo de pacientes com IRA (43 (37–66) mg/dL), como também a creatinina (1,00 (0,85–1,35)). Ambas foram significativamente diferentes entre os grupos, assim como o pH (valor de p = 0,001 *versus* 0,03). Hematócrito e hemoglobina foram significativamente reduzidos no grupo com IRA (valor de p < 0,001 *versus* 0,001). Os demais parâmetros bioquímicos e hemodinâmicos mostraram-se similares e na faixa tolerável de normalidade durante o período evolutivo dos pacientes (Tabela 2).

**Tabela 2:** Parâmetros hemodinâmicos e bioquímicos dos pacientes com e sem injúria renal aguda – Brasília, DF, Brasil, 2021–2023.

Características	Sem LRA (n = 51)	Com LRA (n = 23)	Total (n = 74)	Valor de p
Ureia média (mg/dL) mediana (IIQ)	32 (30–36)	43 (37–66)	35 (30–44)	0,001
pH médio mediana (IIQ)	7,37 (7,33–7,40)	7,39 (7,36–7,42)	7,37 (7,34–7,40)	0,03
Lactato médio(mg/dL) mediana (IIQ)	26,8 (21,5–39,0)	22,1 (14,8–31,5)	25,8 (18,1–35,0)	0,1
Potássio médio (mEq/L) mediana (IIQ)	4,3 (4,1–4,5)	4,1 (4,0–4,4)	4,2 (4,0–4,4)	0,1
Sódio médio (mEq/L) mediana (IIQ)	139 (138–140)	139 (138–143)	139 (138–141)	0,09
PAM (mmHg) mediana (IIQ)	95 (90–98)	98 (92–103)	96 (90–101)	0,07
Frequência cardíaca média (bpm) mediana (IIQ)	98 (93–104)	103 (95–111)	99 (93–106)	0,1
Frequência respiratória média (ipm) mediana (IIQ)	20,3 (18,9–21,8)	20,7 (19,9–22,4)	20,5 (19,0–22,0)	0,3
SpO2 médio (%) mediana (IIQ)	96,6 (95,6–96,9)	96,8 (95,6–97,3)	96,6 (95,6–97,0)	0,4
Creatinina sérica mediana (IIQ)	0,87 (0,76–1,01)	1,00 (0,85–1,35)	0,90 (0,78–1,05)	0,046
Hb médio (g/dL) mediana (IIQ)	10,4 (9,7–11,0)	9,4 (9,0–10,1)	10,1 (9,2–10,7)	<0,001
Ht médio (%) mediana (IIQ)	30,5 (28,4–32,5)	27,7 (26,3–29,9)	29,8 (27,5–31,8)	0,001
Glicemia médio (mg/dL) mediana (IIQ)	187 (156–224)	192 (165–230)	188 (160–225)	0,4
Plaqueta média (μL) mediana (IIQ)	199000 (174000–245000)	217000 (169000–263000)	205000 (170000–255000)	0,7

Legenda: teste U de Mann-Whitney; Hb – hemoglobina; Ht – hematócrito; PAM – pressão arterial média; IRA – injúria renal aguda; IIQ – intervalo interquartil; SpO2 – saturação de oxigênio.

Conforme evidenciado na Tabela 3, independentemente do grupo, o sexo masculino foi predominante. A idade mediana acima de 60 anos caracterizou os dois grupos, assim como o sobrepeso e a etnia preta ou parda, ainda que sem diferença estatística. Em mais da metade de ambos os grupos, o motivo de internação foi cirúrgico. Antibióticos betalactâmicos, como cefuroxima, cefazolina e piperacilina, foram usados de forma significativa. O tempo de permanência na UTI foi significativamente superior no grupo com IRA (valor de p = 0,02), assim como o tempo de CEC (valor de p = 0,04). O SAPS 3 e o SOFA foram significativamente alterados no grupo com IRA (valor de p 0,02 *versus* 0,003).

**Tabela 3:** Associação de variáveis clínicas com a presença ou ausência de injúria renal aguda – Brasília, DF, Brasil, 2021–2023.

Características	Sem LRA (n = 51)	Com LRA (n = 23)	Valor de p
Sexo masculino	41 (80,4)	16 (69,6)	0,3
Idade (anos) mediana (IIQ)	63 (55–65)	64 (61–70)	0,1
IMC (kg/m2) mediana (IIQ)	26,5 (24,7–28,3)	26,9 (23,6–29,6)	0,9
Etnia			
Preta ou parda n (%)	37 (72,5)	14 (60,9)	0,1
Comorbidades n (%)			
Diabetes mellitus	19 (37,3)	13 (56,5)	0,2
Hipertensão arterial sistêmica	36 (70,6)	19 (82,6)	0,3
Doença respiratória	3 (5,9)	5 (21,7)	0,06
Doença vascular periférica	2 (4,0)	2 (8,7)	0,6
Doença vascular cerebral	1 (2,0)	1 (4,3)	0,5
Medicamentos n (%)			
Catecolamina	50 (98,0)	23 (100,0)	0,9
Adrenalina	4 (7,8)	5 (21,7)	0,1
Dobutamina	49 (96,1)	23 (100,0)	0,9
Noradrenalina	40 (78,4)	20 (87,0)	0,5
Vasopressina	11 (21,6)	4 (17,4)	0,8
Vasodilatadores	35 (68,6)	13 (56,5)	0,3
Cefazolina	32 (66,7)	19 (90,5)	0,03
Cefuroxima	16 (31,4)	2 (8,7)	0,03
Meropenem	2 (3,9)	4 (17,4)	0,07
Piperacilina	4 (7,8)	7 (30,4)	0,01
Polimixina	0 (0,0)	1 (4,3)	0,3
ATB glicopeptídeo	2 (3,9)	7 (30,4)	0,003
ATB aminoglicosideo	1 (2,0)	3 (13,0)	0,09
ATB antifúngico	0 (0,0)	1 (4,3)	0,3
Usou diurético	20 (39,2)	13 (56,5)	0,2
TSR n (%)	0 (0,0)	3 (13,0)	0,07
Desfecho da UTI n (%)			
Alta para enfermaria	48 (94,1)	22 (95,7)	0,9
Desfecho final n (%)			
Alta para casa	34 (66,7)	11 (47,8)	0,1
Tempo de UTI (dias) mediana (IIQ)	6 (5–8)	8 (6–16)	0,02
Tempo de hospital (dias) mediana (IIQ)	16 (12–32)	22 (16–35)	0,2
Tempo de cirurgia (minutos) mediana (IIQ)	330 (300–360)	360 (315–405)	0,2
Tempo de CEC (minutos) mediana (IIQ)	101 (80–120)	112 (101–142)	0,04
SAPS3 (admissão) mediana (IIQ)	48 (37–57)	58 (48–64)	0,02
SOFA máximo mediana (IIQ)	6 (5–7)	7 (6–9)	0,003

Legenda: teste exato de Fisher; teste qui-quadrado; teste U de Mann-Whitney; ATB – antibiótico; CEC – circulação extracorpórea; IMC – Índice de Massa Corporal; IRA – injúria renal aguda; UTI – Unidade de Terapia Intensiva; IIQ – intervalo interquartil; TSR – terapia de substituição renal.

A Figura 1 mostra que aumentar o tempo de permanência hospitalar aumenta também a gravidade do paciente, conforme escore SAPS 3.

**Figura 1:** Correlação entre o escore SAPS 3 e tempo de internação hospitalar (Rho = 0,402; valor de p = 0,002). Brasília, DF, Brasil, 2021-2023.

A Figura 2 mostra que tanto o SAPS 3 quanto o SOFA se destacaram pela associação significativa com a IRA, com boa área sob a curva de 0,688 *versus* 0,737.

**Figura 2:** Correlação entre os escores SAPS 3 e SOFA e injúria renal aguda. Brasília, DF, Brasil, 2021–2023.

## DISCUSSÃO

Os resultados deste estudo mostraram que, no pós-operatório de revascularização do miocárdio com CEC, aproximadamente um em cada três pacientes evoluiu com IRA de gravidade leve e moderada (KDIGO 1 e 2). O impacto dos escores SAPS 3 e SOFA na avaliação clínica do grupo com IRA mostrou elevada gravidade, se comparado ao grupo sem IRA, ainda que a mortalidade intra-hospitalar tenha acometido aproximadamente um em cada 12 pacientes e os desfechos da UTI e hospitalar predominantes tenham sido a alta, corroborado pela pequena necessidade de TSR e pela permanência de vários indicadores bioquímicos e hemodinâmicos, em geral, dentro da normalidade na população estudada, apesar do tempo de internação ser prolongado e associado a maior gravidade clínica e consequente risco de morte a longo prazo.

Resultados de evidências científicas demonstraram que, mesmo na IRA leve e reversível, como evidenciado na presente investigação, há risco contínuo de dano tecidual e orgânico e que, *a priori*, a IRA grave pode representar uma diminuição irreversível da função renal e, inclusive, insuficiência renal em estágio terminal^(13,14)^. Estudos mostram maior probabilidade de IRA em idosos com insuficiência cardíaca e histórico de hipertensão, diabetes e outras doenças, consoante aos achados deste estudo. Altas expressões de nitrogênio ureico no sangue e de Crs, além de baixa expressão da TFG estimada, indicam função renal comprometida após cirurgia e, portanto, maior risco de IRA, ainda que de gravidade leve e moderada^(14)^.

Ainda que a idade média dos pacientes com IRA globalmente seja de 60 anos, com a diminuição do *status* socioeconômico, essa idade pode ser reduzida para em torno de 50 anos em países de baixa e média renda. Neste estudo, a idade média dos pacientes com IRA foi superior ao grupo sem IRA, sugerindo que a idade avançada aumenta o risco de IRA após CRM^(15,16)^, corroborado o fato de a maioria ser do sexo masculino, haja vista que a evidência científica destaca que 60% dos pacientes com IRA são homens^(16,17)^.

A CRM com CEC tem sido associada ao aumento significativo dos custos totais, custos pós-operatórios, tempo de internação na UTI e internação hospitalar pós-operatória. Assim, prever o estado de saúde futuro desses pacientes é crucial. Identificar mudanças em sua situação clínica o mais precoce possível pode contribuir para o ajuste do tratamento e, portanto, prevenção da falência de órgãos com resultados desfavoráveis, como o óbito^(18)^. Neste estudo, quanto maior o tempo de internação, maior a gravidade do paciente segundo os escores prognósticos SAPS 3 e SOFA.

Apesar dos avanços científicos, a cirurgia cardiovascular ainda envolve risco para complicações graves no cenário dos cuidados críticos. A IRA representa uma complicação notável, com impacto significativo na evolução clínica dos pacientes, determinante de alta incidência de morbidade e mortalidade^(19)^. Além disso, a IRA requer TSR em 2% a 5% dos pacientes afetados, condição que pode agravar ainda mais a mortalidade^(19)^. Neste estudo, aproximadamente um em cada oito pacientes necessitou de TSR, ainda que a maioria tenha obtido alta hospitalar.

Devido à morbidade e à mortalidade associadas à piora da função renal, o diagnóstico precoce é importante e deve ser buscado em populações de risco^(19)^, como os idosos, também presentes em maior parte deste estudo. Em estudo de coorte retrospectivo realizado com a população da China, as taxas de mortalidade hospitalar para pacientes sem IRA e com IRA estágios 1, 2 e 3 foram de 0%, 2,4%, 10,0% e 22,2%^(15)^, respectivamente, similar aos achados da presente investigação.

Após a cirurgia, a IRA também pode afetar o prognóstico e reduzir a taxa de sobrevivência de longo prazo dos pacientes^(15–16)^. Entre os achados deste estudo, a maioria dos pacientes com IRA no pós-operatório de revascularização com CEC foi identificada precocemente, ou seja, nas primeiras 48 horas de pós-operatório, o que ressalta a importância de alertas eletrônicos em programas laboratoriais como mecanismo disparador para detecção precoce de IRA. Na prática clínica, ao reconhecer alterações na Crs, esses alertas incrementam a notificação e proporcionam iniciativas mais precoces. Com isso, é possível obter melhores resultados para os pacientes, assim como benefícios potenciais durante a hospitalização e em longo prazo^(15,20)^.

Estudo retrospectivo, conduzido com dados de pacientes submetidos à cirurgia de coração aberto na Arábia Saudita, destacou que pacientes com tempos de *bypass* cardiopulmonar (CEC) maiores que 90 minutos apresentaram danos renais mais significativos^(21)^. Os resultados do presente estudo mostraram que o tempo de CEC superior a 100 minutos impactou de forma significativa o comprometimento renal. A CEC pode produzir uma série de fatores inflamatórios e causar danos aos tecidos. O suporte prolongado da CEC pode aumentar a hemólise, a liberação de hemoglobina livre e danificar a função renal ao liberar ferro como uma toxina endógena^(15)^.

A CEC isoladamente ou associada ao envelhecimento pode predispor à redução da massa renal, alteração tubular e esclerótica do rim. Esse processo causa redução do fluxo sanguíneo renal e declínio da TFG a uma taxa de 6,3 mL/min/1,73 m^2^ por década. Portanto, a capacidade fisiológica diminuída do rim aumenta o risco de IRA^(19)^. Neste estudo, a amostra foi caracterizada principalmente por pacientes idosos, ainda que a idade isoladamente não tenha sido um fator significativo para ocorrência da IRA.

Revisão sistemática de 13 estudos evidenciou que o tempo mediano de permanência na UTI de pacientes com IRA pelo critério de creatinina do KDIGO foi de 0,75 a 10 dias^(22)^, semelhante aos achados do presente estudo, em que a mediana do tempo de internação nesse grupo de pacientes foi de oito dias.

A CRM impõe a necessidade de transfusão intraoperatória de hemácias, conforme destacado em estudo de coorte retrospectiva^(15)^. No presente estudo, foi possível identificar diferença significativa nos níveis de hemoglobina entre pacientes com e sem IRA. Taxas diminuídas de hemoglobina são fatores determinantes de transfusão^(23)^. Tanto a anemia quanto a transfusão de hemácias podem aumentar o risco de IRA perioperatória. A anemia causa aumento do estresse hipóxico renal e necessidade de transfusão de hemácias por aumento da resposta inflamatória e lesão relacionada aos radicais livres^(23)^.

Meta-análise de ensaios clínicos constatou que pacientes com IRA são caracterizados por perda rápida da função renal, o que pode desencadear distúrbios eletrolíticos, acidose metabólica, sobrecarga de fluidos e aumento de toxinas urêmicas séricas^(24)^. A uremia compreende um processo patológico que acompanha a IRA^(24)^, como identificado entre os pacientes com IRA neste estudo. As doenças renais e cardíacas estão inter-relacionadas, e em ambos os distúrbios, verificam-se vários fatores de risco comuns, como hipertensão arterial e diabetes mellitus, também reconhecidos entre os achados deste estudo como as comorbidades mais frequentes.

O estabelecimento de escores prognósticos, como SOFA e SAPS 3, com base em funções sistêmicas de múltiplos órgãos, é uma estratégia de análise da gravidade prognóstica de pacientes críticos e uma das melhores formas de dirimir os riscos, subsidiando o cuidado centrado no paciente^(25)^. É amplamente utilizado em terapia intensiva para nortear decisões clínicas e cuidados assistenciais^(26)^. Conforme evidenciado nestes achados, com boa área sob a curva, escores prognósticos se mostraram confiáveis preditores da IRA no pós-operatório de revascularização do miocárdio com CEC.

Revisão sistemática incluindo 15 estudos concluiu que a toxicidade renal foi maior em pacientes em uso de vancomicina associado à piperacilina-tazobactam do que com qualquer outro agente isoladamente ou vancomicina associada a meropenem ou cefepima. Os presentes achados evidenciaram que antibióticos betalactâmicos, como os descritos, foram significativamente utilizados em pacientes que evoluíram com IRA no pós-operatório de CRM. A utilização de fármacos nefrotóxicos é um fator de risco modificável para IRA, e contribui em torno de 18,2% a 48,7% dos casos de IRA em pacientes críticos, em que os antibióticos atuam como gatilhos essenciais para IRA^(27,28)^.

As opções de tratamento para IRA são muito limitadas. O diagnóstico clínico e o reconhecimento de sinais da IRA por profissionais como o enfermeiro podem contribuir para a implementação de medidas preventivas adequadas, baseadas em sistemas de pontuação/escores prognósticos, o que pode efetivamente favorecer a recuperação precoce da IRA, ainda que o diagnóstico dessa síndrome seja clinicamente tardio e norteado pela Crs.

As limitações deste estudo resultam da abordagem unicêntrica, com risco de viés de aferição relacionado ao registro de dados em prontuários, tamanho amostral limitado e emprego somente do critério de creatinina da classificação KDIGO, para avaliação da função renal, em razão da imprecisão do registro de volume urinário.

## CONCLUSÃO

Escores prognósticos como SAPS 3 e SOFA mostraram bom desempenho e evidenciaram que o grupo com IRA foi clinicamente de maior gravidade. Entre os pacientes em pós-operatório de CRM, aproximadamente um em cada três evoluiu com IRA de gravidade leve e moderada (KDIGO 1 e 2) e maior tempo de permanência tanto na UTI quanto na unidade hospitalar.

# Translation: Impacto de los puntajes pronósticos en la evaluación de la lesión renal aguda en el postoperatorio de revascularización miocárdica

**DOI:** 10.1590/1980-220X-REEUSP-2024-0410es

**Authors:** Abraão Alves dos Reis, Tayse Tâmara da Paixão Duarte, Michelle Zampieri Ipolito, Kamilla Grasielle Nunes da Silva, Paulo Percio Mota Magro, Marcia Cristina da Silva Magro

**Affiliations:** 1 Universidade de Brasília, Faculdade de Ciências e Tecnologias em Saúde, Brasília, DF, Brasil.; 2 Hospital Universitário de Brasília, Brasília, DF, Brasil.; 3 Instituto Federal de Brasília, Brasília, DF, Brasil.

**Keywords:** Cuidados Críticos, Puntuación Fisiológica Simplificada Aguda, Lesión Renal Aguda, Revascularización Miocárdica

## Abstract

**Objetivo:**

Determinar el impacto del Simplified Acute Physiology Score 3 y el Sequential Organ Failure Assessment en la evaluación de la gravedad de pacientes con lesión renal aguda en el postoperatorio de revascularización miocárdica con circulación extracorpórea.

**Método:**

Estudio de cohorte retrospectivo, con muestra no probabilística. El análisis inferencial se realizó mediante las pruebas chi-cuadrado de Pearson, exacta de Fisher y Mann-Whitney, con un nivel de significancia del 5%.

**Resultados:**

La prevalencia de lesión renal aguda fue del 31,4%. El Simplified Acute Physiology Score 3 y el Sequential Organ Failure Assessment mostraron puntuaciones más altas en pacientes con lesión renal (58 (48–64) versus 48 (37–57), p = 0,02; 7 (6–9) versus 6 (5–7), p = 0,003), además de una estancia más prolongada en cuidados intensivos, 8 (6–16) versus 6 (5–8) (p = 0,02) días, respectivamente.

**Conclusión:**

Los pacientes con lesión renal aguda permanecieron más tiempo en cuidados intensivos, y el Simplified Acute Physiology Score 3 y el Sequential Organ Failure Assessment mostraron buen desempeño, mostrando mayor gravedad entre los pacientes con lesión renal aguda en el postoperatorio de la cirugía de revascularización miocárdica.

## INTRODUCCIÓN

La lesión renal aguda ( LRA) es una complicación tras la cirugía cardiaca con incidencias de entre el 5% y el 42%, dependiendo de la gravedad, los criterios diagnósticos utilizados y las características del paciente, que afecta a los resultados a corto y largo plazo^(1)^. La gravedad de la LRA es el principal determinante de la mortalidad a corto y largo plazo, con tasas del 36,67% y el 64,66%(2). Además, la LRA se asocia a un aumento de la duración de la estancia hospitalaria y de los costes sanitarios totales^(3)^.

La fisiopatología de la LRA asociada a la cirugía cardiaca aún no se conoce del todo. En general, las causas multifactoriales predisponen a su aparición durante los periodos preoperatorio, intraoperatorio y postoperatorio, ya que implica complejas interacciones entre los factores de riesgo del paciente, las características de la cirugía y los acontecimientos clínicos posteriores^(4,5)^. Estos factores actúan de forma sinérgica y contribuyen al desarrollo de diferentes mecanismos de lesión renal. Por lo tanto, es crucial la asistencia de precisión, basada en la predicción y la personalización de estrategias centradas en el paciente, aplicadas precisamente en el momento adecuado durante el curso de la gestión clínica, para lograr mejores resultados clínicos^(3)^. Esto incluye la identificación precoz de factores de riesgo individuales, como comorbilidades preexistentes y parámetros de laboratorio alterados, así como la adopción de intervenciones específicas^(4)^.

El bypass aortocoronario para revascularización predomina entre los procedimientos de cirugía cardiaca con circulación extracorpórea (CEC). Presenta un alto riesgo de desarrollar LRA, aunque los distintos abordajes intervencionistas influyen en la aparición de LRA^(4)^. Este riesgo surge de la compleja interacción entre la naturaleza invasiva del procedimiento, los factores fisiológicos implicados en la CEC y las características clínicas de los pacientes^(6)^.

Con el fin de gestionar adecuadamente la LRA asociado a la cirugía cardiaca, se han utilizado modelos de predicción o sistemas de puntuación que, al resultar prometedores en la identificación precoz de los pacientes en riesgo, facilitan y optimizan las estrategias de tratamiento en el postoperatorio y, por tanto, pueden contribuir a mejorar el pronóstico^(4)^. El pronóstico de los pacientes con LRA depende no sólo de la etiología de la enfermedad que lo ha provocado, sino también de los métodos diagnósticos y del manejo utilizados. El uso de puntuaciones pronósticas, como *la Simplified Acute Physiology Score* 3 (SAPS 3)^(7)^ y la *Sequential Organ Failure Assessment*(SOFA)^(8)^, utilizadas en la Unidad de Cuidados Intensivos (UCI) para predecir el riesgo de mortalidad, permite realizar una estimación razonable del riesgo de muerte para la toma de decisiones clínicas centradas en el paciente, reduciendo la posibilidad de una evolución complicada y peores resultados.

La eficacia de estas puntuaciones pronósticas parece estar bien aceptada en los pacientes de cuidados intensivos, pero aún existe un vacío en cuanto a estudios que muestren la relación entre el SAPS 3 y el SOFA en el periodo post-revascularización coronaria. El rendimiento comparativo de las puntuaciones SAPS 3 y SOFA en la identificación de pacientes con mayor riesgo de resultados desfavorables sigue estando poco explorado. Teniendo esto en cuenta, el objetivo de este estudio es determinar el impacto del uso de las puntuaciones pronósticas SAPS 3 y SOFA en la evaluación de la gravedad de los pacientes con LRA en el período postoperatorio de la revascularización miocárdica con CEC.

## MÉTODO

### Diseño, Período y Lugar del Estudio

Se trata de un estudio de cohortes cuantitativo y retrospectivo basado en la guía *STrengthening the Reporting of OBservational studies in Epidemiology* (STROBE). Se realizó en una UCI de adultos de un hospital público docente de la región Centro-Oeste de Brasil. Los datos se recogieron entre julio de 2023 y junio de 2024.

### Definición de la Muestra y Criterios de Selección

La muestra fue una muestra de conveniencia no probabilística de pacientes sometidos a revascularización miocárdica con CEC. Se incluyeron los pacientes mayores de 18 años y post operados sometidos a revascularización con CEC. Se excluyó a los pacientes que se habían sometido a cirugía combinada, estaban en cuidados paliativos, habían sido reoperados, no había datos suficientes en la historia clínica, tenían una tasa de filtración glomerular (TFG) estimada inferior a 30 ml/min/1,73 m^2^, según la fórmula de *la Chronic Kidney Disease Epidemiology Collaboration* (CKD-EPI)^(9)^, o estaban en tratamiento por enfermedad renal crónica, como diálisis en el periodo previo a la hospitalización o trasplante renal. De los 87 pacientes, diez fueron excluidos por antecedentes de enfermedad renal crónica y tres por información insuficiente para el análisis, constituyendo así una muestra de 74 pacientes.

### Protocolo del Estudio

Se recopilaron datos de pacientes sometidos a revascularización coronaria con CEC entre enero de 2021 y diciembre de 2023. Se realizó un seguimiento de los pacientes durante 15 días desde su ingreso en la UCI, desde el postoperatorio inmediato y cada diez días posteriores hasta el final de la estancia hospitalaria. Para la recogida de datos se utilizó un cuestionario con preguntas estructuradas que contenían ítems sobre caracterización clínica y quirúrgica, puntuaciones pronósticas (SAPS 3 y SOFA), comorbilidades, parámetros de laboratorio, hemodinámicos y quirúrgicos (tiempo de CEC, tiempo de cirugía, transfusión). Los datos se extrajeron de las historias clínicas físicas y electrónicas. Se registró la puntuación SAPS 3 al ingreso en la UCI y la SOFA diaria para la evaluación pronóstica y la identificación de resultados.

Los pacientes incluidos se clasificaron como con LRA y sin LRA en el postoperatorio. La creatinina basal se definió como la creatinina más baja registrada en las historias clínicas entre siete y 365 días antes de la hospitalización. En su ausencia, se registró la creatinina más baja de los siete primeros días de hospitalización^(10)^.

La LRA se definió según la clasificación *Kidney Disease: Improving Global Outcomes* (KDIGO): KDIGO 1 (riesgo de lesión renal), ante un aumento de la creatinina sérica (Crs) de 0,3 mg/dL en 48 horas o un aumento de 1,5 veces o 1,9 veces el valor basal en siete días; KDIGO 2 (lesión renal), cuando el aumento de la Crs era de 2,0 a 2,9 veces el valor basal; y KDIGO 3 (insuficiencia renal), cuando el aumento de la Crs era de 3,0 veces el valor basal o cuando se requería tratamiento renal sustitutivo (TRS). Sólo en los pacientes clasificados en el estadio 3 está indicado iniciar un tratamiento renal sustitutivo. Se clasificaron como sin deterioro renal si la Crs y la TFG previa eran mayores o iguales a 60 ml/min/1,73 m^2(11)^.

Los sistemas de predicción pronóstica SAPS 3 y SOFA han demostrado su utilidad en el ámbito clínico de los pacientes críticos de la UCI. Se utilizó el SAPS 3 porque es una puntuación pronóstica de la gravedad de la enfermedad, con el objetivo de predecir la mortalidad a partir de los datos obtenidos al ingreso. El SOFA se utilizó para identificar la disfunción orgánica, describiendo los cambios fisiológicos por sistema orgánico^(7–8)^. La LRA se consideró precoz cuando el paciente desarrolló LRA en las 48 horas siguientes a su ingreso en la UCI, y tardío cuando el paciente sufrió LRA durante más de 48 horas^(12)^.

### Análisis y Procesamiento de Datos

Los datos se organizaron en hojas de cálculo de *Microsoft Excel 2021* ^®^ y se comprobaron dos veces. El análisis *estadístico* se realizó con el *programa Statistical Package for the Social Sciences* (IBM^®^SPSS^®^), versión 23. Se utilizó el análisis descriptivo para calcular frecuencias absolutas y relativas, media, mediana, desviación estándar e intervalo intercuartílico para describir las características de las variables y proporcionar información resumida sobre los datos recogidos.

Para el análisis inferencial, se aplicó la prueba de normalidad de Kolmogorov-Smirnov, que no mostró una distribución normal. A continuación se llevaron a cabo pruebas no paramétricas, como la prueba chi-cuadrado de Pearson y la prueba exacta de Fisher, para investigar la asociación entre distintas variables categóricas. La prueba de Mann-Whitney se utilizó para comparar las medianas de dos muestras independientes en situaciones en las que los datos no cumplían los supuestos de distribución normal y homogeneidad de varianzas. El nivel de significación adoptado fue del 5%.

### Aspectos Éticos

El estudio se realizó de acuerdo con las directrices éticas nacionales e internacionales y fue aprobado por el Comité de Ética en Investigación Humana de la Facultad de Ciencias y Tecnologías de la Salud de la Universidad de Brasilia, bajo el Certificado de Sometimiento a Evaluación Ética nº 61486022.0.0000.8093 y el Dictamen nº 5.740.310. Todos los pacientes incluidos firmaron el Consentimiento Libre e Informado.

## RESULTADOS

De los 74 pacientes con y sin LRA, predominaron los varones (77,0%) y la etnia parda (67,6%). La edad superior a 60 años era similar en ambos grupos, al igual que el índice de masa corporal superior a 26 kg/m^2^. La mayoría (74,3%) refería hipertensión y diabetes mellitus (43,2%) como comorbilidades. Todos precisaron antibióticos y fármacos vasoactivos, siendo la noradrenalina el más frecuente (81,1%). Además, el 44,6% utilizó diuréticos y el 4,1% recibió TRR.

Casi todos (94,6%) fueron dados de alta de la UCI. La duración media de la estancia en la UCI para el grupo con LRA fue ligeramente superior en comparación con el grupo sin LRA (8 (6–16) frente a 6 (5–8) días), al igual que la duración de la estancia hospitalaria (22 (16–35) frente a 16 (12–32) días). La mediana del tiempo quirúrgico fue superior a 300 minutos, y el tiempo de CEC fue superior a 100 minutos en ambos grupos. La gravedad de los pacientes con LRA representada por el SAPS 3 fue de 58 (48–64), y por el SOFA fue de 7 (6–9), superior a la del grupo sin LRA (Tabla 1).

**Tabla 1:** Características demográficas y clínicas de los pacientes en el postoperatorio de bypass coronario con CEC – Brasilia, DF, Brasil, 2021–2023.

Características	No LRA (n = 51)	Con LRA (n = 23)	Total (n = 74)
Hombre	41 (80,4)	16 (69,6)	57 (77,0)
Edad (años) media ± DE; mediana (IIC)	60 ± 8	64 ± 7	62 ± 8
	63 (55–65)	64 (61–70)	63 (57–67)
Etnia			
Blancos n (%)	4 (7,8)	5 (21,7)	9 (12,2)
Negro n (%)	3 (5,9)	1 (4,3)	4 (5,4)
Amarillo n (%)	1 (2,0)	0 (0,0)	1 (1,4)
Pardo n (%)	37 (72,5)	13 (56,5)	50 (67,6)
No declarado n (%)	7 (13,7)	3 (13,0)	10 (13,5)
Comorbilidades			
Diabetes n (%)	19 (37,3)	13 (56,5)	32 (43,2)
Hipertensión n (%)	36 (70,6)	19 (82,6)	55 (74,3)
Enfermedad respiratoria n (%)	3 (5,9)	5 (21,7)	8 (10,8)
Enfermedad vascular periférica n (%)	2 (4,0)	2 (8,7)	4 (5,4)
Enfermedad cerebrovascular n (%)	1 (2,0)	1 (4,3)	2 (2,7)
Medicamentos n (%)			
Uso de drogas vasoactivas	51 (100,0)	23 (100,0)	74 (100,0)
Adrenalina	4 (7,8)	5 (21,7)	9 (12,2)
Dobutamina	49 (96,1)	23 (100,0)	72 (97,3)
Noradrenalina	40 (78,4)	20 (87,0)	60 (81,1)
Vasopresina	11 (21,6)	4 (17,4)	15 (20,3)
Vasodilatadores	35 (68,6)	13 (56,5)	48 (64,9)
ATB usado	51 (100,0)	23 (100,0)	74 (100,0)
Polimixina	0 (0,0)	1 (4,3)	1 (1,4)
Glucopéptido ATB	2 (3,9)	7 (30,4)	9 (12,2)
Aminoglucósido ATB	1 (2,0)	3 (13,0)	4 (5,4)
Antifúngico ATB	0 (0,0)	1 (4,3)	1 (1,4)
Usó un diurético	20 (39,2)	13 (56,5)	33 (44,6)
Diurético de asa	18 (35,3)	13 (56,5)	31 (41,9)
Diurético ahorrador de potasio	4 (7,8)	2 (8,7)	6 (8,1)
TSR n (%)	0 (0,0)	3 (13,0)	3 (4,1)
Resultado UCI n (%)			
Alta a planta	48 (94,1)	22 (95,7)	70 (94,6)
Alta hospitalaria	2 (4,0)	2 (4,0)	4 (5,4%)
Resultado hospitalario n (%)			
Muerte	1 (2,0)	2 (8,7)	3 (4,1)
Alta a domicilio	34 (66,7)	11 (47,8)	45 (60,8)
Remitido a otro sector	16 (31,4)	8 (34,8)	24 (32,4)
Derivado a otro hospital	0 (0,0)	1 (4,3)	1 (1,4)
Tiempo en UCI (días) mediana (IIC)	6 (5–8)	8 (6-16)	7 (5-9)
Estancia hospitalaria (días) mediana (IIC)	16 (12-32)	22 (16–35)	20 (13–32)
Tiempo de cirugía (minutos) mediana (IIC)	330 (300–360)	360 (315–405)	330 (300–360)
Tiempo de CEC (minutos) mediana (IIC)	101 (80–120)	112 (101–142)	105 (90–120)
SAPS 3 (admisión) mediana (IIC)	48 (37–57)	58 (48–64)	53 (41–60)
Mediana máxima SOFA (IIC)	6 (5–7)	7 (6–9)	6 (5–7)

Leyendas: ATB: antibiótico; CEC: bypass cardiopulmonar; IMC: índice de masa corporal; IRA: lesión renal aguda; SAPS 3: *Simplified Acute Physiology Score (puntuación simplificada de fisiología aguda) 3*; SOFA: *Sequential Organ Failure Assessment (evaluación secuencial del fallo orgánico)*; UCI: unidad de cuidados intensivos; IIC: intervalo intercuartílico; TRR: terapia renal sustitutiva; DE: desviación estándar.

En el grupo de pacientes con LRA predominaban las KDIGO 1 (12,2%) y 2 (12,2%), es decir, los que presentaban riesgo de daño renal. No obstante, más de la mitad presentaban una función renal conservada (68,9%). De los 23 pacientes con LRA, 20 (87,0%) se diagnosticaron precozmente y 3 (13,0%) tardíamente.

La urea era mayor en el grupo de pacientes con LRA (43 (37–66) mg/dL), al igual que la creatinina (1,00 (0,85–1,35)). Ambas eran significativamente diferentes entre los grupos, al igual que el pH (p-valor = 0,001 frente *a* 0,03). El hematocrito y la hemoglobina se redujeron significativamente en el grupo de la LRA (valor de p < 0,001 frente *a* 0,001). Los demás parámetros bioquímicos y hemodinámicos fueron similares y estuvieron dentro del rango normal tolerable durante la evolución de los pacientes (Tabla 2).

**Tabla 2:** Parámetros hemodinámicos y bioquímicos de pacientes con y sin lesión renal aguda – Brasilia, DF, Brasil, 2021–2023.

Características	No LRA (n = 51)	Con LRA (n = 23)	Total (n = 74)	p-valor
Media de urea (mg/dL) mediana (IIC)	32 (30–36)	43 (37–66)	35 (30–44)	0,001
pH medio (IIC)	7,37 (7,33–7,40)	7,39 (7,36–7,42)	7,37 (7,34–7,40)	0,03
Lactato medio (mg/dL) mediana (IIC)	26,8 (21,5–39,0)	22,1 (14,8–31,5)	25,8 (18,1–35,0)	0,1
Media de potasio (mEq/L) mediana (IIC)	4,3 (4,1–4,5)	4,1 (4,0–4,4)	4,2 (4,0–4,4)	0,1
Sodio medio (mEq/L) mediana (IIC)	139 (138–140)	139 (138–143)	139 (138–141)	0,09
PAM (mmHg) mediana (IIC)	95 (90–98)	98 (92–103)	96 (90–101)	0,07
Frecuencia cardiaca media (lpm) mediana (IIC)	98 (93–104)	103 (95–111)	99 (93–106)	0,1
Frecuencia respiratoria media (ipm) mediana (IIC)	20,3 (18,9–21,8)	20,7 (19,9–22,4)	20,5 (19,0–22,0)	0,3
SpO2 media (%) mediana (IIC)	96,6 (95,6–96,9)	96,8 (95,6–97,3)	96,6 (95,6–97,0)	0,4
Creatinina sérica mediana (IIC)	0,87 (0,76–1,01)	1,00 (0,85–1,35)	0,90 (0,78–1,05)	0,046
Hb media (g/dL) mediana (IIC)	10,4 (9,7–11,0)	9,4 (9,0–10,1)	10,1 (9,2–10,7)	<0,001
Ht media (%) mediana (IIC)	30,5 (28,4–32,5)	27,7 (26,3–29,9)	29,8 (27,5–31,8)	0,001
Glucemia media (mg/dL) mediana (IIC)	187 (156–224)	192 (165–230)	188 (160–225)	0,4
Promedio de plaquetas (μL) mediana (IIC)	199000 (174000–245000)	217000 (169000–263000)	205000 (170000–255000)	0,7

Leyenda: prueba U de Mann-Whitney; Hb: hemoglobina; Ht: hematocrito; PAM: presión arterial media; LRA: lesión renal aguda; IIC: Intervalo intercuartílico; SpO2: saturación de oxígeno.

Como se muestra en la Tabla 3, independientemente del grupo, predominaban los varones. Ambos grupos se caracterizaban por una mediana de edad superior a 60 años, así como por el sobrepeso y la etnia negra o parda, aunque no existían diferencias estadísticas. En más de la mitad de ambos grupos, el motivo de la hospitalización fue quirúrgico. Se utilizaron significativamente antibióticos betalactámicos como la cefuroxima, la cefazolina y la piperacilina. La duración de la estancia en la UCI fue significativamente mayor en el grupo de la LRA (p-valor = 0,02), al igual que el tiempo de CEC (p-valor = 0,04). El SAPS 3 y el SOFA se alteraron significativamente en el grupo de LRA (p-valor 0,02 *frente a* 0,003).

**Tabla 3:** Asociación de variables clínicas con la presencia o ausencia de lesión renal aguda – Brasilia, DF, Brasil, 2021–2023.

Características	No LRA (n = 51)	Con LRA (n = 23)	p-valor
Hombre	41 (80,4)	16 (69,6)	0,3
Edad mediana (años) (IIC)	63 (55–65)	64 (61–70)	0,1
IMC (kg/m^2^) mediana (IIC)	26,5 (24,7–28,3)	26,9 (23,6–29,6)	0,9
Etnia			
Negro o pardo n (%)	37 (72,5)	14 (60,9)	0,1
Comorbilidades n (%)			
Diabetes mellitus	19 (37,3)	13 (56,5)	0,2
Hipertensión arterial sistémica	36 (70,6)	19 (82,6)	0,3
Enfermedades respiratorias	3 (5,9)	5 (21,7)	0,06
Enfermedad vascular periférica	2 (4,0)	2 (8,7)	0,6
Enfermedad vascular cerebral	1 (2,0)	1 (4,3)	0,5
Medicamentos n (%)			
Catecolamina	50 (98,0)	23 (100,0)	0,9
Adrenalina	4 (7,8)	5 (21,7)	0,1
Dobutamina	49 (96,1)	23 (100,0)	0,9
Noradrenalina	40 (78,4)	20 (87,0)	0,5
Vasopresina	11 (21,6)	4 (17,4)	0,8
Vasodilatadores	35 (68,6)	13 (56,5)	0,3
Cefazolina	32 (66,7)	19 (90,5)	0,03
Cefuroxima	16 (31,4)	2 (8,7)	0,03
Meropenem	2 (3,9)	4 (17,4)	0,07
Piperacilina	4 (7,8)	7 (30,4)	0,01
Polimixina	0 (0,0)	1 (4,3)	0,3
Glucopéptido ATB	2 (3,9)	7 (30,4)	0,003
Aminoglucósido ATB	1 (2,0)	3 (13,0)	0,09
Antifúngico ATB	0 (0,0)	1 (4,3)	0,3
Usó un diurético	20 (39,2)	13 (56,5)	0,2
TSR n (%)	0 (0,0)	3 (13,0)	0,07
Resultado UCI n (%)			
Alta a planta	48 (94,1)	22 (95,7)	0,9
Resultado final n (%)			
Alta a domicilio	34 (66,7)	11 (47,8)	0,1
Tiempo en UCI (días) mediana (IIC)	6 (5–8)	8 (6–16)	0,02
Estancia hospitalaria (días) mediana (IIC)	16 (12–32)	22 (16–35)	0,2
Tiempo de cirugía (minutos) mediana (IIC)	330 (300–360)	360 (315–405)	0,2
Tiempo de CEC (minutos) mediana (IIC)	101 (80–120)	112 (101–142)	0,04
SAPS3 (admisión) mediana (IIC)	48 (37–57)	58 (48–64)	0,02
Mediana máxima SOFA (IIC)	6 (5–7)	7 (6–9)	0,003

Leyenda: test exacto de Fisher; test chi-cuadrado; test U de Mann-Whitney; ATB: antibiótico; CEC: bypass cardiopulmonar; IMC: Índice de Masa Corporal; LRA: lesión renal aguda; UCI: Unidad de Cuidados Intensivos; IIC: Intervalo intercuartílico; TRR: terapia renal sustitutiva.

La Figura 1 muestra que el aumento de la duración de la estancia hospitalaria también incrementa la gravedad del paciente, según la puntuación SAPS 3.

**Figura 1:** Correlación entre la puntuación SAPS 3 y la duración de la estancia hospitalaria (Rho = 0,402; p-valor = 0,002). Brasilia, DF, Brasil, 2021–2023.

La Figura 2 muestra que tanto el SAPS 3 como el SOFA se destacaron por su asociación significativa con la LRA, con una buena área bajo la curva de 0,688 *versus* 0,737.

**Figura 2:** Correlación entre las puntuaciones SAPS 3 y SOFA y lesión renal aguda. Brasilia, DF, Brasil, 2021–2023.

## DISCUSIÓN

Los resultados de este estudio mostraron que en el postoperatorio de revascularización coronaria con CEC, aproximadamente uno de cada tres pacientes desarrolló LRA de gravedad leve y moderada (KDIGO 1 y 2). La repercusión de las puntuaciones SAPS 3 y SOFA en la evaluación clínica del grupo con FRA mostró una gravedad elevada en comparación con el grupo sin FRA, aunque la mortalidad intrahospitalaria afectó aproximadamente a uno de cada 12 pacientes y los resultados predominantes en la UCI y el hospital fueron el alta, Esto se vio corroborado por la escasa necesidad de TRR y por el hecho de que varios indicadores bioquímicos y hemodinámicos se encontraban en general dentro del rango normal en la población estudiada, a pesar de que la duración de la estancia hospitalaria fue prolongada y se asoció a una mayor gravedad clínica y al consiguiente riesgo de muerte a largo plazo.

Los resultados de la evidencia científica han demostrado que incluso en la LRA leve y reversible, como se puso de manifiesto en esta investigación, existe un riesgo continuo de daño tisular y orgánico y que, a priori, la LRA grave puede representar una disminución irreversible de la función renal e incluso insuficiencia renal terminal^(13,14)^. Los estudios han demostrado una mayor probabilidad de LRA en ancianos con insuficiencia cardiaca y antecedentes de hipertensión, diabetes y otras enfermedades, en consonancia con los hallazgos de este estudio. Las expresiones elevadas de nitrógeno ureico en sangre y de Crs, así como la baja expresión de la TFG estimada, indican un deterioro de la función renal tras la cirugía y, por tanto, un mayor riesgo de FRA, aunque sea de gravedad leve o moderada^(14)^.

Aunque la edad media de los pacientes con LRA en todo el mundo es de 60 años, a medida que disminuye el nivel socioeconómico esta edad puede reducirse a unos 50 años en los países de ingresos bajos y medios. En este estudio, la edad media de los pacientes con IRA fue superior a la del grupo sin IRA, lo que sugiere que la edad avanzada aumenta el riesgo de IRA tras revascularización coronaria^(15,16)^, corroborado por el hecho de que la mayoría eran varones, dado que la evidencia científica destaca que el 60% de los pacientes con IRA son hombres^(16,17)^.

La revascularización coronaria con CEC se ha asociado a un aumento significativo de los costes totales, los costes postoperatorios, la duración de la estancia en la UCI y la estancia hospitalaria postoperatoria. Por lo tanto, es crucial predecir el futuro estado de salud de estos pacientes. Identificar los cambios en su situación clínica lo antes posible puede ayudar a ajustar el tratamiento y, por tanto, a prevenir un fallo orgánico con resultados desfavorables como la muerte^(18)^. En este estudio, cuanto más prolongada era la estancia hospitalaria, mayor era la gravedad del paciente según las puntuaciones pronósticas SAPS 3 y SOFA.

A pesar de los avances científicos, la cirugía cardiovascular sigue implicando un riesgo de complicaciones graves en el contexto de los cuidados críticos. La LRA es una complicación notable, con un impacto significativo en la evolución clínica de los pacientes y una alta incidencia de morbilidad y mortalidad^(19)^. Además, la LRA requiere TRR en el 2% al 5% de los pacientes afectados, una situación que puede agravar aún más la mortalidad^(19)^. En este estudio, aproximadamente uno de cada ocho pacientes precisó TRR, aunque la mayoría recibió el alta hospitalaria.

Debido a la morbilidad y la mortalidad asociadas al empeoramiento de la función renal, el diagnóstico precoz es importante y debe buscarse en poblaciones de riesgo^(19)^, como los ancianos, que también estuvieron presentes en la mayor parte de este estudio. En un estudio de cohortes retrospectivo realizado con población china, las tasas de mortalidad hospitalaria de los pacientes sin LRA y con LRA en estadios 1, 2 y 3 fueron del 0%, el 2,4%, el 10,0% y el 22,2%^(15)^, respectivamente, similares a los hallazgos de la presente investigación.

Tras la cirugía, la LRA también puede afectar al pronóstico y reducir la tasa de supervivencia a largo plazo de los pacientes^(15,16)^. Entre los hallazgos de este estudio, la mayoría de los pacientes con IRA en el postoperatorio de revascularización con CEC fueron identificados precozmente, es decir, dentro de las primeras 48 horas postoperatorias, lo que pone de relieve la importancia de las alertas electrónicas en los programas de laboratorio como mecanismo de activación para la detección precoz del IRA. En la práctica clínica, al reconocer alteraciones en los Crs, estas alertas aumentan la notificación y proporcionan iniciativas más tempranas. Esto puede conducir a mejores resultados para los pacientes, así como a beneficios potenciales durante la hospitalización y a largo plazo^(15,20)^.

Un estudio retrospectivo realizado con datos de pacientes sometidos a cirugía a corazón abierto en Arabia Saudí puso de relieve que los pacientes con tiempos de circulación extracorpórea ( CEC ) superiores a 90 minutos presentaban un daño renal más significativo^(21)^. Los resultados de este estudio mostraron que los tiempos de CEC superiores a 100 minutos tenían una repercusión significativa en el deterioro renal. La CEC puede producir una serie de factores inflamatorios y causar daño tisular. La CEC prolongada puede aumentar la hemólisis, la liberación de hemoglobina libre y dañar la función renal al liberar hierro como toxina endógena^(15)^.

La CEC sola o en combinación con el envejecimiento puede predisponer a una reducción de la masa renal y a cambios tubulares y escleróticos en el riñón. Este proceso provoca una reducción del flujo sanguíneo renal y un descenso del FG a un ritmo de 6,3 ml/min/1,73m^2^ por década. Por lo tanto, la disminución de la capacidad fisiológica del riñón aumenta el riesgo de LRA^(19)^. En este estudio, la muestra se caracterizó principalmente por pacientes de edad avanzada, aunque la edad por sí sola no fue un factor significativo en la aparición de la LRA.

Una revisión sistemática de 13 estudios demostró que la mediana de la estancia en la UCI de los pacientes con IRA según el criterio KDIGO de creatinina se situaba entre 0,75 y 10 días^(22)^, de forma similar a los resultados del presente estudio, en el que la mediana de la estancia en este grupo de pacientes fue de ocho días.

La revascularización coronaria impone la necesidad de transfusión intraoperatoria de hematíes, como se puso de relieve en un estudio de cohortes retrospectivo^(15)^. En el presente estudio, fue posible identificar una diferencia significativa en los niveles de hemoglobina entre los pacientes con y sin LRA. La disminución de los niveles de hemoglobina es un factor determinante para la transfusión^(23)^. Tanto la anemia como la transfusión de hematíes pueden aumentar el riesgo de LRA perioperatoria. La anemia provoca un aumento del estrés hipóxico renal y de la necesidad de transfusión de hematíes debido al incremento de la respuesta inflamatoria y de los daños relacionados con los radicales libres^(23)^.

Un metaanálisis de ensayos clínicos reveló que los pacientes con LRA se caracterizan por una rápida pérdida de la función renal, que puede desencadenar alteraciones electrolíticas, acidosis metabólica, sobrecarga de líquidos y un aumento de las toxinas urémicas séricas^(24)^. La uremia es un proceso patológico que acompaña a la LRA^(24)^, como se identificó entre los pacientes con LRA de este estudio. Las enfermedades renales y cardiacas están interrelacionadas, y en ambos trastornos existen varios factores de riesgo comunes, como la hipertensión y la diabetes mellitus, también reconocidas entre los hallazgos de este estudio como las comorbilidades más frecuentes.

El establecimiento de puntuaciones pronósticas, como SOFA y SAPS 3, basadas en las funciones sistémicas de múltiples órganos, es una estrategia para analizar la gravedad pronóstica de los pacientes críticos y una de las mejores formas de reducir los riesgos, apoyando una atención centrada en el paciente^(25)^. Se utiliza ampliamente en cuidados intensivos para orientar las decisiones clínicas y los cuidados^(26)^. Como muestran estos resultados, con una buena área bajo la curva, las puntuaciones pronósticas demostraron ser predictores fiables de la LRA en el postoperatorio de la revascularización coronaria con CEC.

Una revisión sistemática que incluyó 15 estudios concluyó que la toxicidad renal era mayor en los pacientes que tomaban vancomicina asociada a piperacilina-tazobactam que con cualquier otro agente solo o vancomicina asociada a meropenem o cefepime. Los presentes hallazgos muestran que los antibióticos betalactámicos como los descritos se utilizaron de forma significativa en pacientes que desarrollaron LRA en el periodo post- revascularización coronaria. El uso de fármacos nefrotóxicos es un factor de riesgo modificable para el LRA y contribuye a entre el 18,2% y el 48,7% de los casos de LRA en pacientes críticos, en los que los antibióticos actúan como desencadenantes esenciales de LRA^(27,28)^.

Las opciones de tratamiento de la LRA son muy limitadas. El diagnóstico clínico y el reconocimiento de los signos de IRA por parte de profesionales como el personal de enfermería pueden contribuir a la aplicación de medidas preventivas adecuadas, basadas en sistemas de puntuación/puntuación pronóstica, que pueden favorecer eficazmente la recuperación precoz del IRA, aunque el diagnóstico de este síndrome sea clínicamente tardío y esté guiado por la RSC.

Las limitaciones de este estudio se derivan del enfoque unicéntrico, con un riesgo de sesgo de medición relacionado con el registro de los datos en las historias clínicas, el tamaño limitado de la muestra y el uso únicamente del criterio de creatinina de la clasificación KDIGO para evaluar la función renal, debido a la imprecisión del registro del volumen urinario.

## CONCLUSIÓN

Las puntuaciones pronósticas como SAPS 3 y SOFA obtuvieron buenos resultados y mostraron que el grupo con LRA era clínicamente más grave. Entre los pacientes post-revascularización coronaria, aproximadamente uno de cada tres evolucionó con LRA de gravedad leve y moderada (KDIGO 1 y 2) y una mayor duración de la estancia tanto en la UCI como en la unidad hospitalaria.
